# Lipid accumulation product: A novel marker for gout and hyperuricemia

**DOI:** 10.1371/journal.pone.0324139

**Published:** 2025-05-20

**Authors:** Dexian Xian, Wen Wang, Hui Li, Geran Song, Daozheng Xu, Fengjun Zhang, Zhe Wang, Wenchang Xu, Hongyan Meng, Min Peng

**Affiliations:** 1 The First Clinical Medical College, Shandong University of Traditional Chinese Medicine, Jinan, China; 2 Department of Pediatrics, Shanghai Municipal Hospital of Traditional Chinese Medicine, Shanghai University of Traditional Chinese Medicine, Shanghai, China; 3 Department of Acupuncture and Moxibustion, Longhua Hospital, Shanghai University of Traditional Chinese Medicine, Shanghai, China; 4 School of Basic Medical Science, Shandong University, Jinan, China; 5 Department of sleep medicine, Binzhou Traditional Chinese Medicine Hospital, Binzhou, China; 6 Department of Pulmonary Diseases, ShuGuang Hospital Affiliated to Shanghai University of Traditional Chinese Medicine, Shanghai, China; 7 College of Acupuncture and Massage, Shandong University of Traditional Chinese Medicine, Jinan, China; 8 Department of Traditional Chinese Medicine, Shandong Provincial Hospital affiliated to Shandong First Medical University, Jinan, Shandong, China; Guangdong Nephrotic Drug Engineering Technology Research Center, Institute of Consun Co. for Chinese Medicine in Kidney Diseases, CHINA

## Abstract

**Background and Aim:**

Using cross-sectional data from the 2009-2018 National Health and Nutrition Examination Survey, the purpose of this study was to investigate the potential link between lipid accumulation product and gout/hyperuricemia

**Methods:**

The data from 10,871 individuals who participated in the National Health and Nutrition Examination Survey spanning the years 2009–2018 were utilized for additional scrutiny. Participants self-reported gout and hyperuricemia as measured by laboratory test data, and other relevant variables and information for LAP were included. Multivariate logistic regression, restricted cubic spline and *p*-trend test were employed to determine the association between LAP and gout/hyperuricemia.

**Results:**

The study included 10,871 adults. The prevalence of hyperuricemia and gout was 20.9% and 5.57%, respectively. Compared with the first quartile, the fourth quartile of lipid accumulation product was associated with a 271% higher risk of hyperuricemia (OR = 3.711, 95% CI 2.732–5.042, *p* < 0.001) in a fully adjusted model. A similar association was found between continuous increase in lipid accumulation product and hyperuricemia (OR = 2.441, 95%CI = 1.348–4.42, *p* = 0.005), with *p* trends showing both < 0.001. The RCS model suggests a significant non-linear relationship between lipid accumulation product and the risk of gout/hyperuricemia. There was an inverted U-shaped relationship between lipid accumulation product and gout/hyperuricemia.

**Conclusions:**

This study confirmed that lipid accumulation product has a high potential to predict the risk of gout/hyperuricemia. These findings suggested that the adjustment of the degree of fat accumulation may be a potential way to prevent and control the onset of gout/hyperuricemia.

## Introduction

Gout is a clinically common inflammatory arthritis characterized by the deposition of sodium urate crystals in joints and soft tissues due to hyperuricemia or elevated levels of uric acid in the blood [1,2]. Hyperuricemia is caused by excessive production of uric acid and insufficient excretion of uric acid due to abnormal kidneys [3]. Globally, the prevalence of gout was 1–4%, the incidence was 0.1–0.3% [4] and hyperuricemia prevalence was 21% [5], with rates in the U.S. ranging between 14.6% and 20% [6]. The increasing and high incidence of gout and hyperuricemia has created a heavy economic burden and a major public health challenge for healthcare systems around the world. Epidemiological studies have highlighted the increasing incidence and prevalence of gout, particularly among older adults and certain demographic groups, underscoring the need for effective management strategies [7,8]. Current treatment modalities primarily focus on pharmacological interventions aimed at lowering uric acid levels and managing acute flares [2,9]; however, these approaches often exhibit limitations, including inadequate response rates, adverse effects, and the potential for long-term complications. Consequently, there is an urgent clinical need for innovative and efficient diagnostic and therapeutic strategies to address the growing challenge posed by gout and hyperuricemia, thereby improving patient outcomes and alleviating the associated societal burden.

Obesity is a multifaceted condition influenced by various risk factors, including genetic predisposition [10], lifestyle choices [11], and environmental influences [12]. Previous studies have established a strong correlation between obesity and numerous health complications, such as cardiovascular diseases and metabolic syndrome, underscoring the critical need for effective assessment tools [13,14]. Traditional metrics for evaluating obesity, such as Body Mass Index (BMI) and Waist Circumference (WC), have been widely utilized; however, they exhibit significant limitations in accurately reflecting the complex nature of adiposity and its associated health risks [15,16]. In this context, the novel Lipid Accumulation Product (LAP), which integrates WC and fasting triglycerides (TG), emerges as a promising alternative [17]. This innovative metric not only enhances the precision of obesity assessment but also serves as a potential indicator for cardiovascular diseases [18] and metabolic syndrome [19], thereby highlighting the substantial implications of this research in advancing our understanding of obesity-related health outcomes.

Despite existing studies from various countries and regions on the correlation between LAP and hyperuricemia, such as Liu et al.‘s finding through the China Health and Retirement Longitudinal Study (CHARLS) that LAP is significantly associated with hyperuricemia [20], Zhou et al.’s large-scale prospective cohort study revealing a positive correlation between LAP and the risk of hyperuricemia in central China [21] and Korean scholars’ research indicating a positive association between hyperuricemia and LAP in the Korean population [22], the reliability of the relevant evidence is partial. To date, no comprehensive study has conclusively demonstrated the correlation between LAP and gout/hyperuricemia.

This study employs a comprehensive epidemiological approach to investigate the relationship between LAP and the incidence of gout and hyperuricemia within the general population of the United States. The advantage of this methodology lies in its ability to integrate diverse demographic and clinical data, thereby enhancing the robustness of the findings and allowing for a more nuanced understanding of the associations at play. The primary objective of this research is to elucidate the potential correlation between LAP and the risk of developing gout and hyperuricemia, addressing the existing gaps in the literature and providing a more definitive assessment of this relationship. By focusing on a well-defined population and employing rigorous statistical analyses, this study aims to contribute valuable insights that may inform clinical practices and public health strategies related to metabolic disorders.

## Materials and methods

### Data source and study population

**Fig 1:**
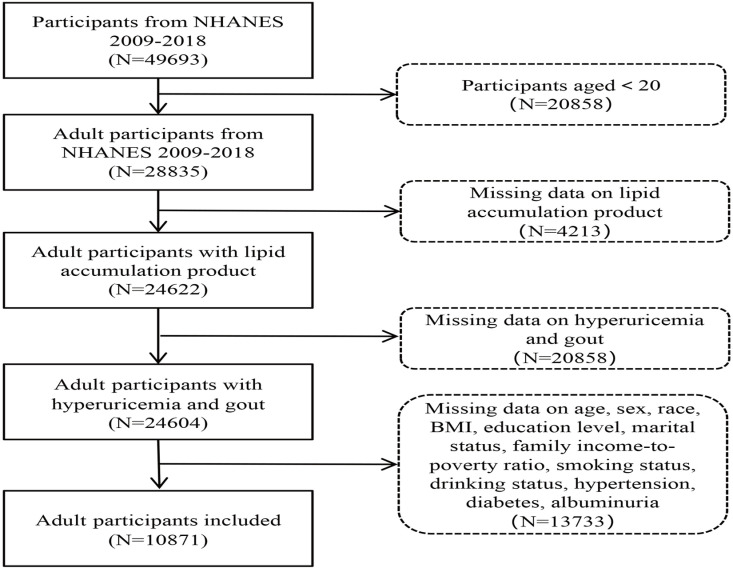
Flowchart of the study. NHANES, the National health and nutrition examination survey.

The National Health and Nutrition Examination Survey (NHANES) is a cross-sectional study conducted by the National Center for Health Statistics (NCHS) of the U.S. Centers for Disease Control and Prevention (CDC) to investigate and confirm the nutrition and health status of the U.S. population. The data was analyzed over a span of five cycles, ranging from the year 2009–2018 (S1 Fig). All participants in the research voluntarily completed informed consent forms, and the study plan received approval from the NCHS. For more details on specific ethical considerations, please visit the website (https://www.cdc.gov/nchs/nhanes/irba98.htm), under NHANES-NCHS Research Ethics Review Board. Exclusion criteria were as follows: the subjects were younger than 20 years old, and the test data omitted LAP data, high uric acid data, and gout diagnosis. The detailed information was shown in the flowchart, as shown in Fig 1.

### Evaluation of lipid accumulation product

The process of measuring triglyceride levels in the NHANES study involves collecting fasting blood samples at mobile testing facilities by healthcare professionals. These samples are then analyzed using the Beckman Synchron LX System, following the U.S. endpoint method. This procedure is detailed in the NHANES 2005–2006 Standard Biochemistry Procedures Data Documentation Codebook and Frequencies publication. The circumference of the waist is gauged by a health professional utilizing a measuring tape, positioned above the top of the hip bones in line with the armpit’s midpoint, at the conclusion of the individual’s regular breath cycle, with a precision of up to one-tenth of a centimeter.

LAP computation formula is as follows: the male LAP = (WC [cm] - 65) × (TG concentration [mmol/L]); Female LAP = (WC [cm] - 58) × (TG concentration [mmol/L]) [23].

### Identification and determination of gout and hyperuricemia

A serum sample of a subject is taken by a professional during a home visit and immediately stored at -30 °C until transported for further analysis. SUA concentrations were quantified as part of the serum biochemical profile using the timed endpoint method of Beckman Coulter UniCel®dx800. According to previous findings, elevated levels of uric acid, termed as hyperuricemia, were characterized by concentrations surpassing 7.0 mg/dL in males and 6.0 mg/dL in females [24,25]. The disease status of gout was determined by the medical Condition questionnaire.

### Covariate

This study considered the following covariates that may have an impact on gout and hyperuricemia, Include Age, Sex, Race, BMI, Education level, poverty-to-income ratio (PIR), marital status, smoking status, Alcohol status, Hypertension, Diabetes, Albuminuria.

BMI was classified as Underweight (BMI less than 18.5), Normalweight (18.5 < BMI < 25), Overweight (25 < BMI < 30), and Obese (BMI > 30). Smoking status was measured by “Have smoked at least 100 cigarettes in your life” and “Do you currently smoke?” The two questions were classified as never smoker (<100 cigarettes in life), former smoker (≥100 cigarettes but no current smoker), and current smoker (≥100 cigarettes and current smoker). Alcohol consumption status was classified by the question “frequency of drinking in the past 12 months” as never drinking, 1–4 drinks per week, and>4 drinks per week. The diagnosis of Hypertension was made based on self-reported questionnaire information or measurements of systolic blood pressure≥140 mmHg and/or diastolic blood pressure≥90 mmHg [26]. The diagnostic basis of diabetes:(1) self-reported diagnosis; (2) Use insulin or diabetes drugs; (3) 2-hour oral glucose tolerance test (OGTT) blood glucose≥200 mg/ dL; (4) Fasting blood glucose≥126 mg/dL; (5) Glycosylated hemoglobin (HbA1c) ≥6.5% [27]. Proteinuria is defined as albumin-to-creatinine ratio (ACR) higher than 30mg/g [28].

### Statistical analysis

In accordance with the CDC guidelines, all statistical analyses were performed. Mean ± standard error is used for continuous variables, while categorical variables are expressed as n (%). The LAP data were classified by quartile, with the lowest quartile as the reference category, and the trend *p* was calculated.

The correlation between LAP and hyperuricemia\gout was examined through the application of multivariate logistic regression, with results expressed as 95% confidence intervals (CI) and odds ratios (ORs). We employed three distinct models to conduct the inquiry: Model 1, as the original model, did not account for any possible confounding elements; Model 2 was based on model 1, and adjusted for basic variables such as age, sex, race, BMI, education level, marital status, family income, smoking and drinking. Based on the adjustment of Model 2, Model 3 further adjusted for metabolism-related variables such as hypertension, diabetes, and albuminuria.

Since LAP is not linearly related to hyperuricemia and gout, the nonlinear relationship between LAP and gout is analyzed using restricted cubic splines. The statistical analysis was conducted using R 4.3.0. Statistical significance was determined with a p value of 0.05 for all bilateral tests.

## Results

### Study participant characteristics

In this study, 10,871 adult participants were selected from the NHANES database from 2009 to 2018. During the previous data inclusion and exclusion process, we excluded 20,858 participants younger than 20 years old, and subsequently 17,964 adult participants with missing LAP, hyperuricemia indicators, and gout diagnosis. The study showed that 606 (5.57%) adult participants had gout and 10,265 (94.4%) adult participants did not have gout. As depicted in Table 1, a total of 2,273 adults accounted for 20.91% with hyperuricemia, whereas the remaining 8,598 individuals, representing 79.09%, were without this condition. The demographic and lifestyle factors, including age, sex, race, BMI, marital status, smoking status, drinking status, hypertension status, diabetes status and proteinuria, exhibited substantial variations when comparing individuals with LAP to those with hyperuricemia and gout (*p* < 0.05).

**Table 1:** Baseline characteristics of subjects.

Characteristics	Overall	Hyperuricemia		p-value	Overall	Gout		p-value
		No	Yes			No	Yes	
**N**	10871	8598	2273		10871	10265	606	
**LAP**	70.72	63.85	99.04	<0.001	70.72	69.24	100.63	<0.001
**LAP_quartile**				<0.001				<0.001
Q1	1,817	1,690	127		1,817	1,782	35	
Q2	2,769	2,363	406		2,769	2,661	108	
Q3	3,114	2,412	702		3,114	2,923	191	
Q4	3,171	2,133	1,038		3,171	2,899	272	
**Age (years)**	48.80	48.24	51.10	<0.001	48.80	48.21	60.56	<0.001
**Sex, %**				<0.001				<0.001
Female	4,715	3,896	819		4,715	4,577	138	
Male	6,156	4,702	1,454		6,156	5,688	468	
**Race, %**				<0.001				0.013
Mexican American	1,367	1,146	221		1,367	1,327	40	
Other Hispanic	935	775	160		935	898	37	
Non-Hispanic White	5,100	4,064	1,036		5,100	4,789	311	
Non-Hispanic Black	2,249	1,658	591		2,249	2,101	148	
Other Race	1,220	955	265		1,220	1,150	70	
**BMI (kg/m2)**	29.01	28.27	32.09	<0.001	29.01	28.89	31.53	<0.001
**BMI_status**				<0.001				<0.001
Underweight	143	131	12		143	137	6	
Normalweight	2,894	2,590	304		2,894	2,805	89	
Overweight	3,680	2,974	706		3,680	3,478	202	
Obese	4,154	2,903	1,251		4,154	3,845	309	
**Education level, %**				0.2				0.2
Low Education	2,176	1,729	447		2,176	2,040	136	
Medium Education	2,544	1,976	568		2,544	2,381	163	
High Education	6,151	4,893	1,258		6,151	5,844	307	
**Marital status, %**				0.007				<0.001
Never married	1,869	1,509	360		1,869	1,828	41	
Married or Living with partner	6,511	5,207	1,304		6,511	6,110	401	
Previously married	2,491	1,882	609		2,491	2,327	164	
**PIR**	3.19	3.20	3.17	0.5	3.19	3.19	3.23	0.7
**Smoking status**				0.001				<0.001
Never smoker	5,158	4,143	1,015		5,158	4,955	203	
Former smoker	3,242	2,428	814		3,242	2,956	286	
Current smoker	2,471	2,027	444		2,471	2,354	117	
**Drinking status**				<0.001				<0.001
No Alcohol	3,524	2,747	777		3,524	3,274	250	
1-4 drinks per week	5,475	4,422	1,053		5,475	5,261	214	
>4 drinks per week	1,872	1,429	443		1,872	1,730	142	
**Hypertension**				<0.001				<0.001
Non-hypertensive	4,918	4,116	802		4,918	4,742	176	
Hypertensive	5,953	4,482	1,471		5,953	5,523	430	
**Diabetes**				<0.001				<0.001
Borderline	3,883	2,961	922		3,883	3,685	198	
Diabetic	2,000	1,446	554		2,000	1,769	231	
Non-Diabetic	4,988	4,191	797		4,988	4,811	177	
**Albuminuria**				<0.001				<0.001
Normal	9,520	7,663	1,857		9,520	9,072	448	
High	1,351	935	416		1,351	1,193	158	

*LAP* lipid accumulation product, *BMI* Body Mass Index, *PIR* poverty-to-income ratio.

### Relationship between LAP index and gout/hyperuricemia

We analyzed the quartile distribution of the LAP index in the NHANES data, and plotted the density curve between Quartiles (Q1-Q4). The results showed that the quartile range of LAP was Q1(0.334–20.784), Q2(20.835–43.371), Q3(43.404–81.102), Q4(81.123–1468.545), and the data showed an obvious left-skewed distribution trend. Lower LAP values (mainly concentrated in Q1 and Q2) have higher density, while the density gradually decreases with the increase of LAP value, and the distribution of LAP values in Q3 and Q4 is more dispersed.

Multivariate logistic regression analysis was performed to clarify the relationship between LAP and gout. According to the present study, in the continuity model adjusted for basal and metabolic variables, the risk of gout increased by about 0.02% for each additional unit of LAP (OR=1.002, 95%CI 1–1.003, *p* < 0.001). To further verify this association, we performed a quartile analysis of LAP as a categorical variable, which showed that the risk of gout disease also increased. After full adjustment, the odds of developing gout were significantly increased by approximately 144.1% in the highest LAP quartile array (Q4) compared to the lowest group (Q1) (OR=2.441, 95%CI 1.348–4.42, *p* < 0.001) (Table 2).

**Table 2:** Association between the LAP and gout/hyperuricemia risk among adults in NHANES 2009–2018.

	Model 1 OR (95%CI) *p*-value	Model 2 OR (95%CI) *p*-value	Model 3 OR (95%CI) *p*-value
**Gout**			
**Continuous in LAP**	1.004 (1.002, 1.005) **<0.001**	1.002 (1.001, 1.003) **0.003**	1.002 (1, 1.003) **0.012**
**Quartiles of LAP**			
Q1(0.334–20.784)	Reference	Reference	Reference
Q2(20.835–43.371)	2.561 (1.373, 4.776) **0.004**	1.481 (0.794, 2.761) **0.222**	1.518 (0.807, 2.855) **0.201**
Q3(43.404–81.102)	4.302 (2.581, 7.17) **<0.001**	2.039 (1.215, 3.421) **0.009**	2.06 (1.207, 3.517) **0.011**
Q4(81.123–1468.545)	6.324 (3.776, 10.593) **<0.001**	2.519 (1.421, 4.464) **0.002**	2.441 (1.348, 4.42) **0.005**
***p*** for trend	**<0.001**	**<0.001**	**<0.001**
**Hyperuricemia**			
**Continuous in LAP**	1.006 (1.005, 1.007) **<0.001**	1.003 (1.002, 1.004) **<0.001**	1.003 (1.002, 1.004) **<0.001**
**Quartiles of LAP**			
Q1(0.334–20.784)	Reference	Reference	Reference
Q2(20.835–43.371)	2.513 (1.906, 3.314) **<0.001**	1.957 (1.465, 2.613) **<0.001**	1.936 (1.443, 2.596) **<0.001**
Q3(43.404–81.102)	3.781 (2.945, 4.853) **<0.001**	2.41 (1.793, 3.239) **<0.001**	2.35 (1.739, 3.174) **<0.001**
Q4(81.123–1468.545)	7.105 (5.71, 8.84) **<0.001**	3.8 (2.818, 5.124) **<0.001**	3.711 (2.732, 5.042) **<0.001**
***p*** for trend	**<0.001**	**<0.001**	**<0.001**

Model 1: Non-adjusted

Model 2, Adjusted for age, sex, race, BMI, education level, marital status, family income, smoking and drinking.

Model 3, Model 2 plus additional adjustment for hypertension, diabetes, and albuminuria

*OR* Odd ratio, *CI* Confidence intervals, *LAP* lipid accumulation product

LAP index showed a positive correlation with hyperuricemia, and two different models were adjusted to take into account a range of health behaviors and metabolic variables such as hypertension, diabetes, and proteinuria. The findings indicated a positive correlation between LAP and hyperuricemia risk across both models. In a comprehensively adjusted continuous variable model, the risk of hyperuricemia increased by 0.3% for each additional unit of LAP (OR = 1.003, 95%CI: 1.002–1.004, *p* < 0.001). The analysis of LAP using quartiles revealed that the group in Q4 had a significantly increased risk of hyperuricemia, which was 2.711 times higher compared to those in Q1 (OR: 3.711; 95% CI: 2.732–5.042; p < 0.001) (Table 2).

### Nonlinear relationship between LAP index and gout/hyperuricemia

**Fig 2:**
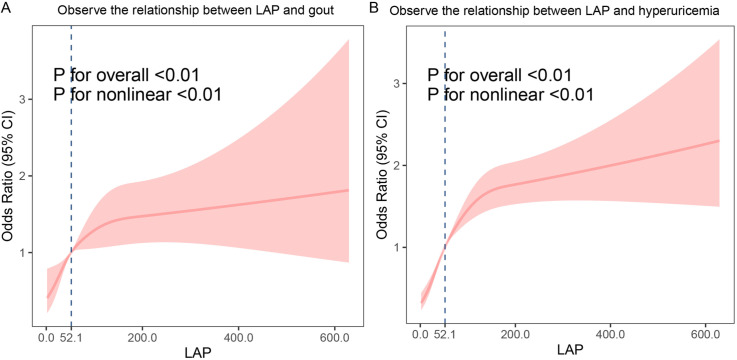
Nonlinear relationship between LAP and the risk of gout/hyperuricemia A, LAP and gout; B, LAP and hyperuricemia.

RCS was used to estimate the nonlinear association between LAP and gout/hyperuricemia. The results showed that the relationship between LAP index measured by RCS and gout/hyperuricemia was non-linear (Fig 2), and hyperuricemia and gout increased with the increase of LAP level. There was an inverted U-shaped relationship between LAP and gout/hyperuricemia.

## Discussion

Based on the data from the NHANES database, this is the first comprehensive cross-sectional examination of LAP and gout/hyperuricemia. The study analyzed health data from a representative U.S. population of adults from 2009 to 2018. The results from multivariate logistic regression analysis revealed a positive association between LAP levels and the incidence of gout or hyperuricemia. Additionally, RCS analysis showed a significant non-linear relationship between LAP and the incidence of gout/hyperuricemia. These findings strongly support the predictive value of LAP in the onset of gout/hyperuricemia.

In this study, the prevalence of gout in the NHANES database was 5.57%, while the prevalence of hyperuricemia was 20.91%. In previous studies, the combined prevalence of gout and hyperuricemia in China was 1.1% and 13.3 [29], respectively. The incidence of gout and hyperuricemia in Australia was 5.2% and 16.6% respectively [30]; The incidence of gout and hyperuricemia in Korea was 0.75% and 11.4%, respectively [31,32]. These results suggest that the incidence of gout and hyperuricemia varies by country and ethnicity, which may be related to ethnicity and the diet and lifestyle of the population [33], which is also confirmed by our study. As key contributors to the prevalence of cardiovascular diseases, they have emerged as a significant concern in public health. Therefore, early detection and management of gout/hyperuricemia through anthropometric indicators before clinical symptoms appear is essential for the management and prevention of metabolic abnormalities such as gout/hyperuricemia.

It is well known that gout and hyperuricemia are positively associated with traditional obesity indices such as body mass index BMI\WWI and body roundness index (BRI) [34]. However, research has shown that the reliability of the traditional obesity index is controversial, i.e., the distribution of fat varies according to the anatomical site [35]. Therefore, in recent years, experts have proposed a new obesity indicator, LAP, which is a combination of WC and TG that better reflects cardiovascular risk and is superior to BMI and WWI in predicting hyperuricemia [23].

In our study, LAP index was positively correlated with gout and hyperuricemia. This is consistent with previous studies in other areas. According to the 2016 NHANES survey report in South Korea, there is a positive correlation between blood uric acid level and LAP in the general population [22]. In a cross-sectional study in central China, LAP was positively correlated with the risk of hyperuricemia and showed a dose-response relationship with gender, age, and eGFR [21]. Chen et al. found that LAP’s ability to predict the risk of hyperuricemia was significantly improved after gender division of the population [36], which was also confirmed by Liu et al. ‘s cohort study in the CHARLS [37], which may be related to the different distribution characteristics of visceral adipose tissue in men and women.

Researches on the relationship between obesity caused by adipose tissue accumulation and uric acid are continuing to deepen worldwide. Obesity has different effects on the production and excretion of uric acid in different populations, and the related pathway mechanism of the two is proposed. Studies have shown that obesity mediates adipose tissue remodeling, which recruits and activates macrophages. Pro-inflammatory factors such as TNF-α, IL-6, and IL-8 are secreted by macrophages [38], while adipose tissue itself secretes anti-inflammatory factors such as IL-4, IL-10, and IL-19 [39], which form an antagonistic equilibrium relationship. However, adipose tissue dysfunction caused by obesity breaks the balance between the secretion of pro-inflammatory factors and anti-inflammatory factors, resulting in loss of homeostasis and chronic low-grade inflammation [40] which in turn leads to abnormal uric acid metabolism [41]. At the same time, a lack of oxygen in obese fat tissue can trigger the production of uric acid. In an animal experiment, Ye Jianping et al. [42] found that adipose tissue hypoxia in obese mice resulted in decreased adiponectin expression and increased inflammatory gene expression, and adiponectin expression level was closely related to hyperuricemia [43]. In obese individuals, the accumulation of uric acid may lead to increased oxidative stress, which further worsens kidney damage [44]. Therefore, understanding the interaction and regulatory mechanism between obesity and uric acid has significant significance for the prevention and treatment of cardiovascular diseases, metabolic syndrome, insulin resistance and other diseases.

However, there are limitations to our study. First of all, the original data of this study came from the NHANSE database, which is a cross-sectional study. The correlation between LAP and hyperuricemia and gout was analyzed. The research results need to be verified by experiments to further explain the mechanism between the two. Secondly, it is difficult to establish a causal relationship between the research results, and it needs to be discussed in combination with studies such as RCT or Mendelian randomization. Third, the diagnosis of gout comes from patient reports, which may present a risk of retrospective bias.

## Conclusions

In conclusion, the results of the present study confirmed the significant positive correlation between LAP and hyperuricemia/gout, suggesting that LAP can be used as a dynamic index for the detection of gout/hyperuricemia. Clinicians would be able to identify people at high risk for early diagnosis of gout/hyperuricemia by LAP, allowing targeted interventions to be implemented to reduce the risk of the latter. However, the specific molecular mechanism of this relationship remains unclear, and future studies should focus on elucidation of the biological link between LAP and hyperuricemia/gout, as well as evaluating the extent to which interventions to regulate LAP index can reduce the incidence of hyperuricemia/gout.

## Supporting information

S1 Fig Dataset year ranges. Exposure data, outcome data and covariate data were extracted from NHANES from 2009 to 2018.
(TIF)

## Abbreviations

LAP: Lipid accumulation product
NHANES: National Health and Nutrition Examination Survey
RCS: restricted cubic spline
BMI: Body Mass Index
WC: Waist Circumference
TG: fasting plasma triglyceride
CHARLS: China Health and Retirement Longitudinal Study
NHANES: National Health and Nutrition Examination Survey
NCHS: National Center for Health Statistics
CDC: U.S. Centers for Disease Control and Prevention
PIR: poverty-to-income ratio
OGTT: oral glucose tolerance test
ACR: albumin-to-creatinine ratio
CI: confidence intervals
ORs: odds ratios
BRI: body roundness index

## References

[1] Dalbeth N, Gosling AL, Gaffo A, Abhishek A. Gout. Lancet. 2021;397(10287):1843–55. doi: 10.1016/S0140-6736(21)00569-9 33798500

[2] Dalbeth N, Choi HK, Joosten LAB, Khanna PP, Matsuo H, Perez-Ruiz F, et al. Gout. Nat Rev Dis Primers. 2019;5(1):69. doi: 10.1038/s41572-019-0115-y 31558729

[3] Lioté F. Hyperuricemia and gout. Curr Rheumatol Rep. 2003;5(3):227–34. doi: 10.1007/s11926-003-0072-y 12744816

[4] Singh JA, Gaffo A. Gout epidemiology and comorbidities. Semin Arthritis Rheum. 2020;50(3S):S11–6. doi: 10.1016/j.semarthrit.2020.04.008 32620196

[5] Fang X-Y, Qi L-W, Chen H-F, Gao P, Zhang Q, Leng R-X, et al. The interaction between dietary fructose and gut microbiota in hyperuricemia and gout. Front Nutr. 2022;9:890730. doi: 10.3389/fnut.2022.890730 35811965 PMC9257186

[6] Chen-Xu M, Yokose C, Rai SK, Pillinger MH, Choi HK. Contemporary prevalence of gout and hyperuricemia in the United States and decadal trends: the National health and nutrition examination survey, 2007-2016. Arthritis Rheumatol. 2019;71(6):991–9. doi: 10.1002/art.40807 30618180 PMC6536335

[7] Kumar M, Manley N, Mikuls TR. Gout flare burden, diagnosis, and management: navigating care in older patients with comorbidity. Drugs Aging. 2021;38(7):545–57. doi: 10.1007/s40266-021-00866-2 34105100

[8] Gerritsen M, Nurmohamed MT. The effects of pharmacological urate-lowering therapy on cardiovascular disease in older adults with gout. Drugs Aging. 2024;41(4):319–28. doi: 10.1007/s40266-024-01098-w 38416394

[9] Richette P, Doherty M, Pascual E, Barskova V, Becce F, Castañeda-Sanabria J, et al. 2016 updated EULAR evidence-based recommendations for the management of gout. Ann Rheum Dis. 2017;76(1):29–42. doi: 10.1136/annrheumdis-2016-209707 27457514

[10] Goodarzi MO. Genetics of obesity: what genetic association studies have taught us about the biology of obesity and its complications. Lancet Diabetes Endocrinol. 2018;6(3):223–36. doi: 10.1016/S2213-8587(17)30200-0 28919064

[11] Reinehr T. Lifestyle intervention in childhood obesity: changes and challenges. Nat Rev Endocrinol. 2013;9(10):607–14. doi: 10.1038/nrendo.2013.149 23897171

[12] Nicolaidis S. Environment and obesity. Metabolism. 2019;100S:153942. doi: 10.1016/j.metabol.2019.07.006 31610854

[13] Moore KJ, Shah R. Introduction to the obesity, metabolic syndrome, and CVD compendium. Circ Res. 2020;126(11):1475–6. doi: 10.1161/CIRCRESAHA.120.317240 32437304 PMC7250157

[14] Saltiel AR, Olefsky JM. Inflammatory mechanisms linking obesity and metabolic disease. J Clin Invest. 2017;127(1):1–4. doi: 10.1172/JCI92035 28045402 PMC5199709

[15] Clark AL, Fonarow GC, Horwich TB. Waist circumference, body mass index, and survival in systolic heart failure: the obesity paradox revisited. J Card Fail. 2011;17(5):374–80. doi: 10.1016/j.cardfail.2011.01.009 21549293

[16] Schetz M, De Jong A, Deane AM, Druml W, Hemelaar P, Pelosi P, et al. Obesity in the critically ill: a narrative review. Intensive Care Med. 2019;45(6):757–69. doi: 10.1007/s00134-019-05594-1 30888440

[17] Kahn HS. The lipid accumulation product is better than BMI for identifying diabetes: a population-based comparison. Diabetes Care. 2006;29(1):151–3. doi: 10.2337/diacare.29.1.151 16373916

[18] Kyrou I, Panagiotakos DB, Kouli G-M, Georgousopoulou E, Chrysohoou C, Tsigos C, et al. Lipid accumulation product in relation to 10-year cardiovascular disease incidence in Caucasian adults: the ATTICA study. Atherosclerosis. 2018;279:10–6. doi: 10.1016/j.atherosclerosis.2018.10.015 30366186

[19] Taverna MJ, Martínez-Larrad MT, Frechtel GD, Serrano-Ríos M. Lipid accumulation product: a powerful marker of metabolic syndrome in healthy population. Eur J Endocrinol. 2011;164(4):559–67. doi: 10.1530/EJE-10-1039 21262912

[20] Liu Y, Zhao W, Liu X, Jiang H, Wu Y, Luo L, et al. Identifying reliable obesity indices for hyperuricemia among middle-aged and elderly populations: a longitudinal study. Lipids Health Dis. 2024;23(1):305. doi: 10.1186/s12944-024-02296-6 39327579 PMC11426091

[21] Zhou W, Shan N, Wei J, Zhou Y, Men M. Cross-sectional and longitudinal associations between lipid accumulation product and hyperuricemia. Nutr Metab Cardiovasc Dis. 2022;32(10):2348–55. doi: 10.1016/j.numecd.2022.06.022 35965249

[22] Seong JM, Park CE, Gi MY, Cha JA, Jung EY, Lee JH, et al. Relationship between uric acid and lipid accumulation product index by gender in Korean adults: the 2016 Korean National health and nutrition examination survey. Prim Care Diabetes. 2021;15(3):541–7. doi: 10.1016/j.pcd.2020.12.001 33358135

[23] Kahn HS. The “lipid accumulation product” performs better than the body mass index for recognizing cardiovascular risk: a population-based comparison. BMC Cardiovasc Disord. 2005;5:26. doi: 10.1186/1471-2261-5-26 16150143 PMC1236917

[24] Feig DI, Kang D-H, Johnson RJ. Uric acid and cardiovascular risk. N Engl J Med. 2008;359(17):1811–21. doi: 10.1056/NEJMra0800885 18946066 PMC2684330

[25] Tan Y, Fu Y, Yao H, Wu X, Yang Z, Zeng H, et al. Relationship between phthalates exposures and hyperuricemia in U.S. general population, a multi-cycle study of NHANES 2007-2016. Sci Total Environ. 2023;859(Pt 1):160208. doi: 10.1016/j.scitotenv.2022.160208 36400295

[26] Whelton PK, Carey RM, Aronow WS, Casey DE, Collins KJ, Dennison HC. ACC/AHA/AAPA/ABC/ACPM/AGS/APhA/ASH/ASPC/NMA/PCNA guideline for the prevention, detection, evaluation, and management of high blood pressure in adults: executive summary: a report of the American college of cardiology/American heart association task force on clinical practice guidelines. J Am Coll Cardiol. 2018;71: 2199–269. doi: 10.1016/j.jacc.2017.11.005 29146533

[27] Scinicariello F, Buser MC, Balluz L, Gehle K, Murray HE, Abadin HG, et al. Perfluoroalkyl acids, hyperuricemia and gout in adults: analyses of NHANES 2009-2014. Chemosphere. 2020;259:127446. doi: 10.1016/j.chemosphere.2020.127446 32590180 PMC8114790

[28] Webster R, Liu W-W, Chaouch A, Lochmüller H, Beeson D. Fast-channel congenital myasthenic syndrome with a novel acetylcholine receptor mutation at the α-ε subunit interface. Neuromuscul Disord. 2014;24(2):143–7. doi: 10.1016/j.nmd.2013.10.009 24295813

[29] Liu R, Han C, Wu D, Xia X, Gu J, Guan H, et al. Prevalence of hyperuricemia and gout in mainland China from 2000 to 2014: a systematic review and meta-analysis. Biomed Res Int. 2015;2015:762820. doi: 10.1155/2015/762820 26640795 PMC4657091

[30] Ting K, Gill TK, Keen H, Tucker GR, Hill CL. Prevalence and associations of gout and hyperuricaemia: results from an Australian population-based study. Intern Med J. 2016;46(5):566–73. doi: 10.1111/imj.13006 26765205

[31] Kim J-W, Kwak SG, Lee H, Kim S-K, Choe J-Y, Park S-H. Prevalence and incidence of gout in Korea: data from the national health claims database 2007-2015. Rheumatol Int. 2017;37(9):1499–506. doi: 10.1007/s00296-017-3768-4 28676911

[32] Kim Y, Kang J, Kim G-T. Prevalence of hyperuricemia and its associated factors in the general Korean population: an analysis of a population-based nationally representative sample. Clin Rheumatol. 2018;37(9):2529–38. doi: 10.1007/s10067-018-4130-2 29790110

[33] Weidong L, Liuting C, Xiangcong C, Jianhong P, Xueying Y. Analysis of the relationship of refractory gout between potential biomarkers and diet structure and lifestyle based on 1H-NMR. J Orthop Surg Res. 2024;19(1):78. doi: 10.1186/s13018-024-04540-2 38243298 PMC10797800

[34] Mao T, He Q, Yang J, Jia L, Xu G. Relationship between gout, hyperuricemia, and obesity-does central obesity play a significant role?-a study based on the NHANES database. Diabetol Metab Syndr. 2024;16(1):24. doi: 10.1186/s13098-024-01268-1 38254222 PMC10804703

[35] Lamarche B. Abdominal obesity and its metabolic complications: implications for the risk of ischaemic heart disease. Coron Artery Dis. 1998;9(8):473–81. doi: 10.1097/00019501-199809080-00002 9847978

[36] Chen D, Lu C, Chen K, Liu T, Li Y, Shan Z, et al. Association between anthropometric indices and hyperuricemia: a nationwide study in China. Clin Rheumatol. 2024;43(3):907–20. doi: 10.1007/s10067-024-06884-w 38315297

[37] Liu Z, Zhou Q, Tang Y, Li J, Chen Q, Yang H, et al. Sex-specific differences in the associations between adiposity indices and incident hyperuricemia among middle-aged and older adults: a nationwide longitudinal study. Front Endocrinol (Lausanne). 2024;15:1336471. doi: 10.3389/fendo.2024.1336471 38405154 PMC10884268

[38] Reilly SM, Saltiel AR. Adapting to obesity with adipose tissue inflammation. Nat Rev Endocrinol. 2017;13(11):633–43. doi: 10.1038/nrendo.2017.90 28799554

[39] Kawai T, Autieri MV, Scalia R. Adipose tissue inflammation and metabolic dysfunction in obesity. Am J Physiol Cell Physiol. 2021;320(3):C375–91. doi: 10.1152/ajpcell.00379.2020 33356944 PMC8294624

[40] Guzik TJ, Skiba DS, Touyz RM, Harrison DG. The role of infiltrating immune cells in dysfunctional adipose tissue. Cardiovasc Res. 2017;113(9):1009–23. doi: 10.1093/cvr/cvx108 28838042 PMC5852626

[41] Diaz-Torne C, Ortiz MA, Garcia-Guillen A, Jeria-Navarro S, Sainz L, Fernandez-Sanchez S, et al. The inflammatory role of silent urate crystal deposition in intercritical gout. Rheumatology (Oxford). 2021;60(11):5463–72. doi: 10.1093/rheumatology/keab335 33839783

[42] Ye J, Gao Z, Yin J, He Q. Hypoxia is a potential risk factor for chronic inflammation and adiponectin reduction in adipose tissue of ob/ob and dietary obese mice. Am J Physiol Endocrinol Metab. 2007;293(4):E1118-28. doi: 10.1152/ajpendo.00435.2007 17666485

[43] Tamba S, Nishizawa H, Funahashi T, Okauchi Y, Ogawa T, Noguchi M, et al. Relationship between the serum uric acid level, visceral fat accumulation and serum adiponectin concentration in Japanese men. Intern Med. 2008;47(13):1175–80. doi: 10.2169/internalmedicine.47.0603 18591837

[44] Zhang M, Cui R, Zhou Y, Ma Y, Jin Y, Gou X, et al. Uric acid accumulation in the kidney triggers mast cell degranulation and aggravates renal oxidative stress. Toxicology. 2023;483:153387. doi: 10.1016/j.tox.2022.153387 36464070

