# Supplementary figures and images for: Lipid accumulation product: A novel marker for gout and hyperuricemia
Source: PLoS One. 2025 May 20;20(5):e0324139. doi: 10.1371/journal.pone.0324139 (PMC12091749; doi:10.1371/journal.pone.0324139)

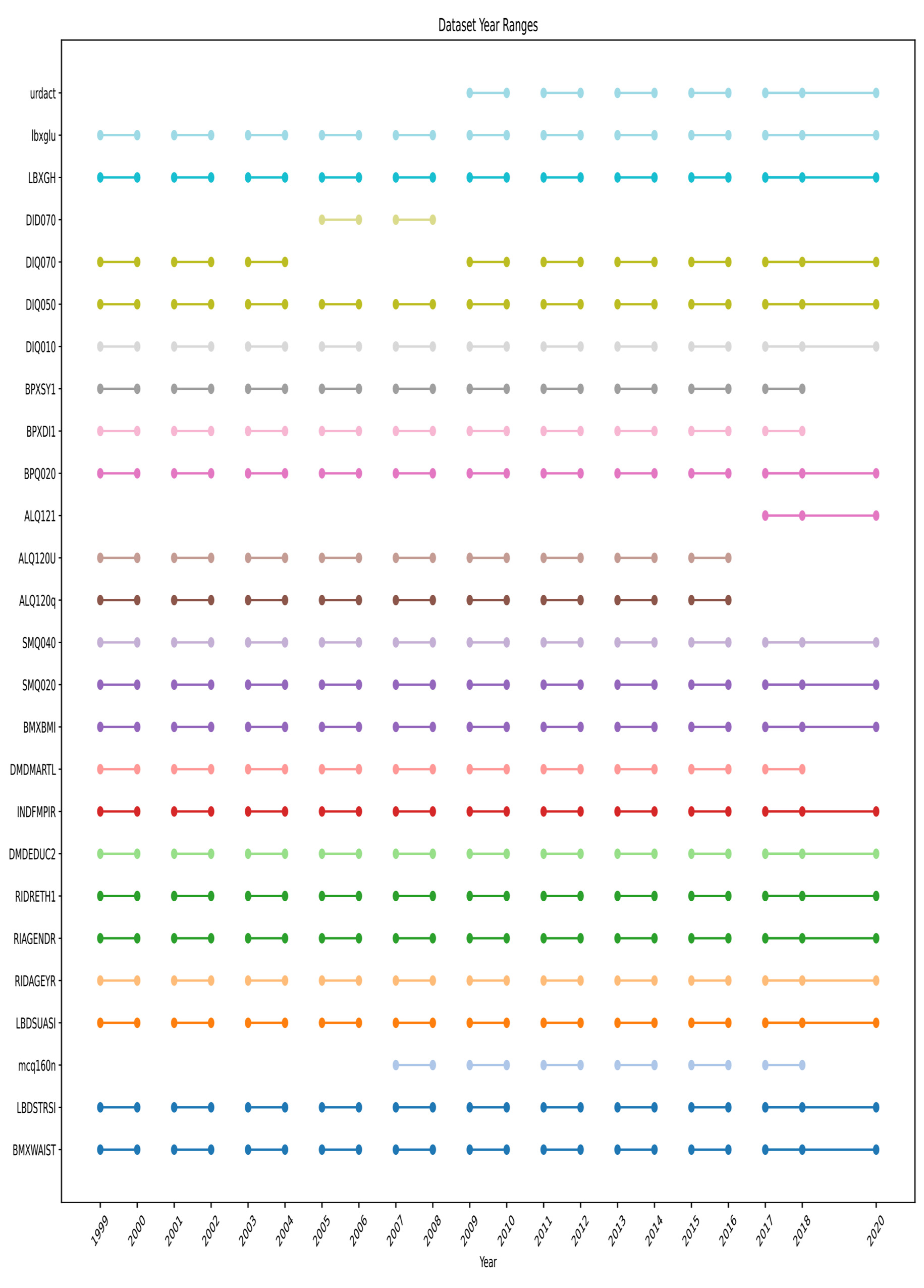

Supplement: S1 Fig — Exposure data, outcome data and covariate data were extracted from NHANES from 2009 to 2018. (TIF) [file pone.0324139.s001.tif]
